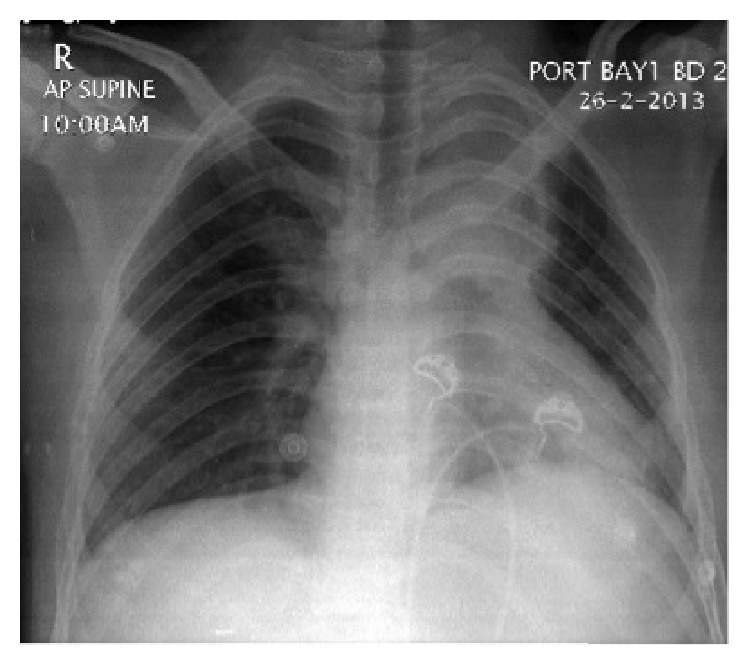# New Onset Refractory Status Epilepticus in a Young Man with H1N1 Infection

**DOI:** 10.1155/2014/585428

**Published:** 2014-10-14

**Authors:** Faisal Ibrahim, Naim Haddad

**Affiliations:** ^1^Division of Neurology “Medicine”, Hamad Medical Corporation, P.O. Box 3050, Doha, Qatar; ^2^Neurology Department, Weill Cornell Medical College in Doha, Doha, Qatar

## Abstract

*Objective*. To report a case of refractory status epilepticus (SE) as an unusual early manifestation of H1N1 influenza infection. *Introduction*. H1N1 neurological complications have been reported and consist mainly of seizures or encephalopathy occurring in children. However, we only found a single report of an adult developing complex partial SE with H1N1 infection. *Case Report*. A 21-year-old previously healthy man was brought to the emergency room (ER) after a witnessed generalized tonic clonic seizure (GTCS). He was fully alert and afebrile upon ER arrival, but a second GTCS prompted treatment with Lorazepam and Fosphenytoin. The initial EEG showed diffuse slowing, but a repeat one requested as the patient failed to regain consciousness revealed recurrent focal seizures of independent bihemispheric origin, fulfilling the criteria for nonconvulsive SE. Chest X-ray, followed by chest CT scan, showed a left upper lobe consolidation. H1N1 infection was confirmed with PCR on bronchoalveolar lavage material. Despite aggressive treatment with Midazolam, Propofol, and multiple high dose antiepileptic drugs, the electrographic seizures recurred at every attempt to reduce the intravenous sedative drugs. The patient died two weeks after his initial presentation. *Conclusion*. H1N1 should be added to the list of rare causes of refractory SE, regardless of the patient's age.

## 1. Introduction

The neurological manifestations of influenza A (H1N1) are well-documented; they mostly consist of seizures and/or encephalopathy, occurring predominantly in young children [1–5]. Status epilepticus (SE) is a rather rare complication of H1N1 infection even at an early age. We only found a single case report of an adult developing complex partial SE with H1N1 infection [6]. Here we report a case of new onset refractory SE as an unusual early and fatal manifestation of H1N1 influenza infection.

## 2. Case Report

A 21-year-old Nepalese man was brought to our emergency room (ER) after a witnessed generalized tonic clonic seizure (GTCS), lasting for few minutes and aborted spontaneously. The patient was previously healthy with no history of seizures. There were no recent respiratory symptoms or fever. His vaccination status is unknown.

**Figure 1:**
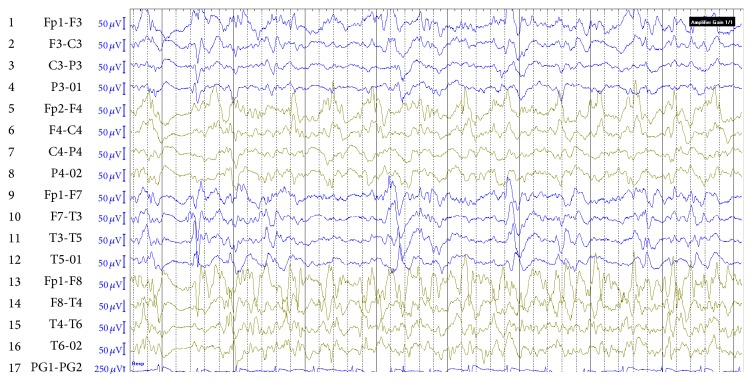


He was fully alert and afebrile with no meningeal signs or focal neurological deficits upon ER arrival. A second GTCS in ER with longer duration prompted treatment with Lorazepam and Fosphenytoin. The patient became subsequently febrile and confused. He desaturated, became hypotensive, and required intubation and ICU admission. The initial EEG showed diffuse generalized slowing with no epileptiform discharges, but a repeat EEG performed on the second day of admission as the patient failed to regain consciousness revealed recurrent focal seizures of independent bihemispheric origin fulfilling the criteria of nonconvulsive SE (Figure 1).

CT head, brain MRI, and cerebrospinal fluid (CSF) analysis were unrevealing. The CSF showed WBC count of two cells/mL, protein of 0.4 g/L, and glucose of 4.4 mmol/L (compared to 5.5 mmol/L serum glucose). CSF cytology, viral screen including herpes, TB PCR and culture, Gram stain, and fungal and bacterial cultures were all negative. Chest X-ray, followed by chest CT scan showed a left upper lobe consolidation (Figure 2). H1N1 infection was confirmed by PCR on bronchoalveolar lavage material. Otherwise, BUN/Creatinine, liver profile, electrolytes, thyroid function tests, RF, ANA, ANCA, HIV serology, and initial blood cultures were unrevealing. Despite aggressive treatment with successive infusions of Midazolam, Propofol and Thiopental, and multiple high doses antiepileptic medications including different combinations of Levetiracetam, Sodium Valproate, Phenytoin, Phenobarbital, and Topiramate, the electrographic seizures recurred at every attempt to reduce the intravenous sedative drugs. The patient was also treated with Oseltamivir. His hospital course was later complicated with septic shock and associated severe hypotension with multiorgan failure, resulting in cardiopulmonary arrest and death two weeks from his initial presentation.

## 3. Discussion

H1N1 infection usually presents with a febrile respiratory illness but it can be a multisystem disease. Seizures and/or encephalopathy have been reported in approximately 7% of H1N1 cases, mainly in the pediatric age group [3, 5, 7]. Glaser et al. reported 44 cases of seizures related to severe H1N1 virus infection, but they did not comment on the occurrence of SE in these patients [2]. In another report, amongst 12 children with seizures complicating H1N1 infection, seven presented with SE [1]. Yildizdaş et al. (2011) reported on eight children with neurological complications; four of them had SE. They also observed full recovery in previously healthy children [8]. Amongst eight patients with H1N1 encephalopathy reported by Fuchigami et al, four had SE [9]. Okumura et al. reported that four of his twenty children with H1N1 encephalopathy had SE, but the seizures were “treated successfully with intravenous antiepileptic drugs” [10]. Other studies reported on single cases of SE in children or adolescents with good recovery [4, 7, 11].

Our case has many unusual features. He is a 21-year-old adult. We only found one well-described case of partial SE with adult H1N1 infection; the reported patient recovered fully within 48 hours [6]. Our patient remained refractory to treatment and ultimately succumbed. Our case also presented with a seizure and ultimately SE without a prodromal febrile respiratory picture; H1N1 was explored and proven after the findings on the chest X-ray.

The pathogenesis of neurological complications of H1N1 infection is unclear. The detection of the virus or its RNA in the CSF of few reported patients would suggest direct viral penetration into the CNS [12]. However, most reported cases lacked CSF pleocytosis, and the viral PCR tested negative in most CSF specimens, suggesting a rather indirect virus-induced inflammatory process. Authors have suggested an overwhelming cytokine release from virus-stimulated glial cells may be responsible for the neurotoxic effects [1, 4, 5, 13, 14].

## 4. Conclusion

H1N1 infection can manifest as SE on initial presentation; this case illustrates the potential for fatal nonconvulsive SE to be caused by H1N1 infection. Therefore, with the company of clinical or radiological respiratory involvement, H1N1 should be added to the list of rare causes of new onset refractory SE regardless of the patient's age.

## Figures and Tables

**Figure 2:**